# Prenatal Maternal Smoke, DNA Methylation, and Multi-omics of Tissues and Child Health

**DOI:** 10.1007/s40572-022-00361-9

**Published:** 2022-06-07

**Authors:** Marta Cosin-Tomas, Ariadna Cilleros-Portet, Sofía Aguilar-Lacasaña, Nora Fernandez-Jimenez, Mariona Bustamante

**Affiliations:** 1 ISGlobal, Institute for Global Health, Barcelona, Spain; 2 Universitat Pompeu Fabra (UPF), Barcelona, Spain; 3 CIBER Epidemiología Y Salud Pública, Madrid, Spain; 4 Department of Genetics, Physical Anthropology and Animal Physiology, University of the Basque Country (UPV/EHU) and Biocruces-Bizkaia Health Research Institute, Basque Country, Spain

**Keywords:** Maternal smoking during pregnancy, Multi-omics, Epigenetics, DNA methylation, Tissue, Child health

## Abstract

**Purpose of Review:**

Maternal tobacco smoking during pregnancy is of public health concern, and understanding the biological mechanisms can help to promote smoking cessation campaigns. This non-systematic review focuses on the effects of maternal smoking during pregnancy on offspring’s epigenome, consistent in chemical modifications of the genome that regulate gene expression.

**Recent Findings:**

Recent meta-analyses of epigenome-wide association studies have shown that maternal smoking during pregnancy is consistently associated with offspring’s DNA methylation changes, both in the placenta and blood. These studies indicate that effects on blood DNA methylation can persist for years, and that the longer the duration of the exposure and the higher the dose, the larger the effects. Hence, DNA methylation scores have been developed to estimate past exposure to maternal smoking during pregnancy as biomarkers.

**Summary:**

There is robust evidence for DNA methylation alterations associated with maternal smoking during pregnancy; however, the role of sex, ethnicity, and genetic background needs further exploration. Moreover, there are no conclusive studies about exposure to low doses or during the preconception period. Similarly, studies on tissues other than the placenta and blood are scarce, and cell-type specificity within tissues needs further investigation. In addition, biological interpretation of DNA methylation findings requires multi-omics data, poorly available in epidemiological settings. Finally, although several mediation analyses link DNA methylation changes with health outcomes, they do not allow causal inference. For this, a combination of data from multiple study designs will be essential in the future to better address this topic.

**Supplementary Information:**

The online version contains supplementary material available at 10.1007/s40572-022-00361-9.

## Introduction

Maternal tobacco smoking during pregnancy (MSDP) is still of great concern in public health. A report including data from 43 countries indicated that the global prevalence of MSDP for the 1985–2016 period was 1.7%, ranging from 0.8% in Africa to 8.1% in Europe [1]. Children of smoker mothers are born with lower birth weight [2]. Moreover, they are at increased risk of developing a long list of pathologies later in life, including obesity [3], impaired lung function and asthma [4], and neuropsychological problems [5].

Before the clinical endpoint, MSDP triggers several molecular, cellular, and physiological pathways in the mother and the offspring. These pathways are diverse due to the number of toxic chemical compounds found in cigarettes and in the smoke produced by their combustion [6]. The epigenome has been proposed as one of the mechanisms linking environmental exposures to disease [7]. It consists of several mitotically heritable chemical modifications of the genome, which control gene expression in a tissue- and time-specific manner, including DNA methylation (DNAm), histone modifications, and regulatory RNAs.

This non-systematic review summarizes currently available literature about the effects of MSDP on the offspring’s epigenome, especially on DNAm, and their link with health outcomes. The review presents what is known about dose and duration of exposure, the persistence of the effects, tissue and cell specificity, and the mediator role of DNAm on health outcomes. Moreover, it presents evidence of the effects on other molecular layers, including gene and microRNA (miRNA) expression and metabolomics. Finally, it discusses the main gaps in the literature and future research directions.

## Maternal Smoking During Pregnancy and Blood DNA Methylation

### Associations with Cord Blood DNA Methylation at Birth

Most epigenome-wide association studies (EWAS) of MSDP assess DNAm through microarrays as they allow evaluating hundreds of participants at a relatively low cost. The most common microarray is the Illumina 450 K, which has since been updated to the MethylationEPIC array, and the most frequently investigated tissue is blood or leukocyte rederived DNA as it is easily accessible in epidemiological settings. On the other hand, information on MSDP is usually obtained through self-reports and less often through objective biomarkers (e.g., urinary cotinine). Obtaining this information at different time points in pregnancy allows to study different exposure windows: any MSDP (when mothers smoke at any time during pregnancy), non-sustained MSDP (when mothers quit smoking at the beginning of pregnancy), and sustained MSDP (when mothers smoke through most of the pregnancy).

The largest EWAS meta-analysis to date evaluating the association of MSDP with cord blood DNAm at birth was conducted by Joubert et al. within the Pregnancy And Childhood Epigenetics (PACE) consortium and included data from 13 cohorts [8••]. After correcting for false discovery rate (FDR), the authors identified 6073 CpGs differentially methylated in relation to sustained MSDP (52% hyper- and 48% hypo-methylated). Consistent with studies of current smoking in adults, the top CpG was cg05575921 located within the *AHRR* gene body, which showed reduced methylation in newborns of smoker mothers (− 6.6%). This gene codes for the protein Aryl hydrocarbon receptor repressor that represses the transcription activity of the Aryl hydrocarbon receptor, a chemical/ligand-dependent intracellular receptor involved in xenobiotic detoxification [9]. Joubert et al. performed pathway enrichment analysis and revealed that genes annotated to CpGs associated with MSDP were enriched for anatomical development, phosphate-containing compound metabolism, nervous system development, and cell communication processes. Among the MSDP-sensitive CpGs, around 30% were common with CpGs found to be associated with own smoking in adults’ blood [10••]. Interestingly, CpGs unique to newborns were enriched in xenobiotic metabolism pathways.

The CpGs most widely identified across other EWAS are situated within coding or regulatory regions of the *AHRR, GFI1*, *CYP1A1*, and *MYO1G* genes (Tables S1). Altered DNAm patterns in these genes were identified in European [11, 12, 13••], Japanese [14], and African American populations [15], suggesting similar effects of MSDP across ancestries. Sex differences in DNAm are frequent and stable throughout early development and are known to alter health risks [16]. Nevertheless, only a few studies have explored sex-specific DNAm patterns associated with MSDP, giving inconsistent results [13••, 15, 17–20]. Furthermore, none of these studies assessed DNAm changes in sex chromosomes, whereas evidences in adult current smokers suggest that they are also present [21].

### Persistence of Associations on Blood DNA Methylation in Childhood, Adolescence, and Adulthood

Literature suggests that environmental exposures may involve lasting alterations in DNAm. Within the PACE meta-analysis, the authors explored whether blood DNAm changes associated with MSDP at birth were still observed in childhood [8••]. They observed that all 6073 CpGs identified in newborns were still nominally associated with MSDP in child blood at a mean age of 5 years (of these, 148 CpGs met FDR significance), and 73% had a consistent direction of effect.

**Fig. 1:**
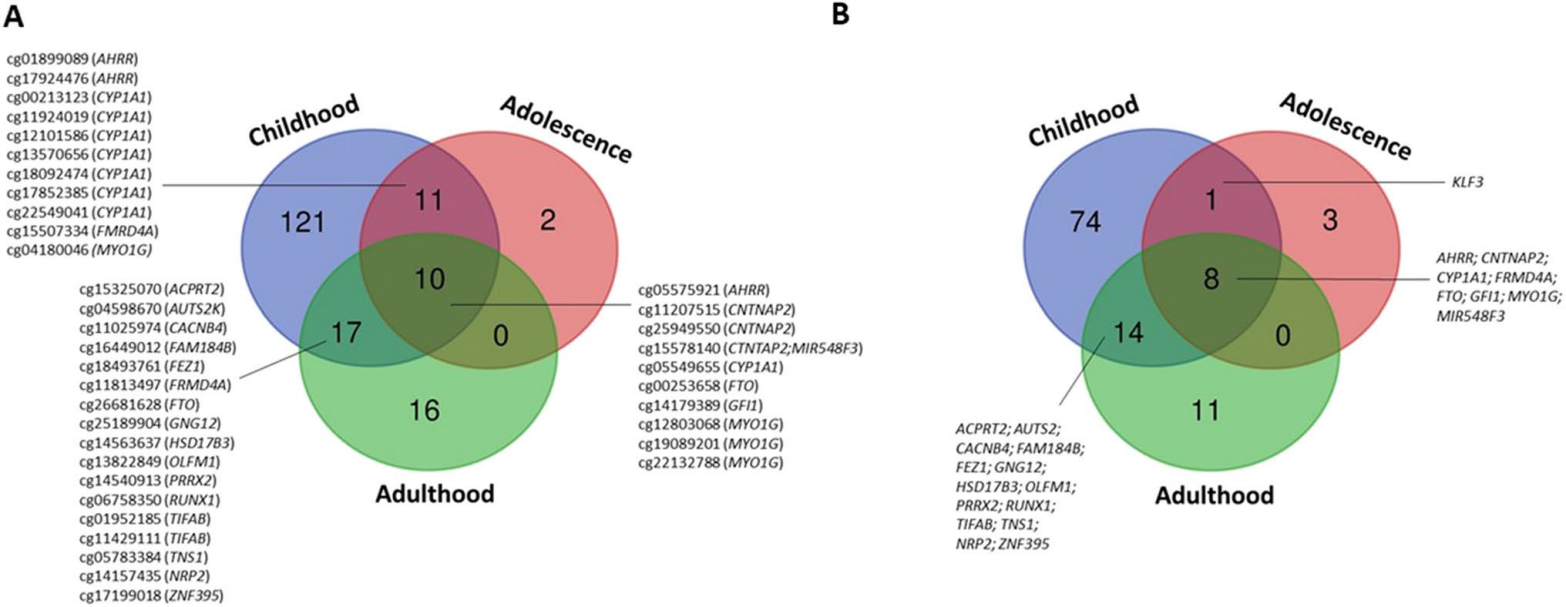
Venn diagram showing the overlap between CpGs (**A**) and genes (**B**) persistently associated with any/sustained maternal smoking during pregnancy in blood in childhood (Richmond et al. [13••]; Joubert et al. [8••]; Vives-Usano et al. [24••]), adolescence (Richmond et al. [13••], Rauschert et al. [20]), and adulthood (Richmond et al. [13••]; Wiklund et al. [26])

Other studies also reported persistent effects of MDSP on blood DNAm after birth, covering different age ranges: infancy [22••], childhood [13••, 23, 24••], adolescence [13••, 20, 25, 26], and adulthood [13••, 20, 26, 27]. The overlap among CpGs and genes showing persistent effects at different ages is shown in Fig. 1.

However, these results are mostly based on evaluations of DNAm levels at only one time-point, which does not allow to examine time-response patterns to MSDP in the same individual. Importantly, the Avon Longitudinal Study of Parents and Children (ALSPAC) cohort measured blood DNAm at birth, 7 and 17 years of age in the same participants. Leveraging this approach, they observed that some CpGs recovered DNAm levels similar to those unexposed (e.g., within *GFI1*, *KLF13*, and *ATP9A* genes), while others showed persistently perturbed DNAm levels throughout childhood and adolescence (e.g., within *AHRR*, *MYO1G*, *CYP1A1*, and *CNTNAP2* genes) [13••].

## Maternal Smoking During Pregnancy and DNA Methylation in Other Tissues

### Associations with Placenta DNA Methylation

Placenta, a critical organ for fetal development, is another accessible tissue in epidemiological settings. It mediates the maternal–fetal exchange of gases, nutrients, hormones, and metabolic waste products, thereby playing a crucial role in shaping fetal growth and birth size, a significant predictor of health across the life course [28]. Despite the importance of this organ for child and adult health, there are limited studies analyzing the effect of environmental exposures on it [29].

The largest study to date investigating placental DNAm was conducted by Everson et al. within the PACE consortium and included data from 7 cohorts [30••]. After Bonferroni correction, the authors identified 443 CpGs differently methylated in response to any or sustained MSDP (41.5% hypo- and 58.5% hyper-methylated). These CpGs were annotated to genes enriched for detoxification pathways, growth-factor signaling, immunity and inflammation, and myometrial and vascular smooth muscle contraction. Moreover, MSDP-associated CpGs were enriched in placental enhancers. The CpG cg27402634, located between *LEKR1* and *LINC00886*, showed the largest effect (− 25.1% in sustained smokers). Besides this, other CpGs that yielded large magnitudes of association but far from the top CpG were located within the *EDC3*, *WBP1L*, and *KDM5B* genes. Interestingly, the genomic regions of *LEKR1-LINC00886*, *EDC3*, and *WBP1L* have been described to contain genetic variants related to birth weight.

These top genes were also identified in other single cohort EWAS by Morales et al. [31] and Cardenas et al. [32]. The latter evaluated DNAm with the Illumina EPIC array, which has a larger coverage of the genome than the 450 K, and this allowed the identification of 52 novel CpGs not described before. Finally, another study identified 203 placental differentially methylated regions (DMRs) associated with MSDP [33•]. These DMRs encompassed 1023 CpGs, some of them overlapping previous literature. They were enriched for placenta enhancer regions located near ten imprinted regions known to control fetal and placental development. Results from other smaller studies are summarized in Table S1.

### Associations with DNA Methylation in Other Tissues

A limited number of studies have investigated the association between MSDP and DNAm in tissues other than blood or placenta (Table S1). Access to fetal tissues is complicated, and thus studies are usually of small size. In dorsolateral prefrontal cortex samples from the second trimester of gestation, 577 DMRs were associated with MSDP at nominal significance (*n* = 24) [34••]. Top DMRs were within the promoter regions of *GNA15* and *SDHAP3*, previously reported to show altered DNAm levels in the brain of patients with autism spectrum disorder and schizophrenia. In fetal lung, MSDP was nominally associated with DNAm at CpGs annotated to several genes, including *DPP10* previously related to asthma (*n* = 85) [35•, 36]. As indicated before, *AHRR* is a primary response gene for MSDP. However, unlike in cord blood, it was not affected in buccal epithelium cells collected at birth (*n* = 15) [18]. Finally, in child buccal epithelium cells (*n* = 272), one study found that MSDP was associated with DNAm differences at eight genes (*AXL*, *PTPRO*, *KLK11*, *TGFB3*, *MET*, *SPDEF*, *SNCG*, *NBL1*) [37].

**Fig. 2:**
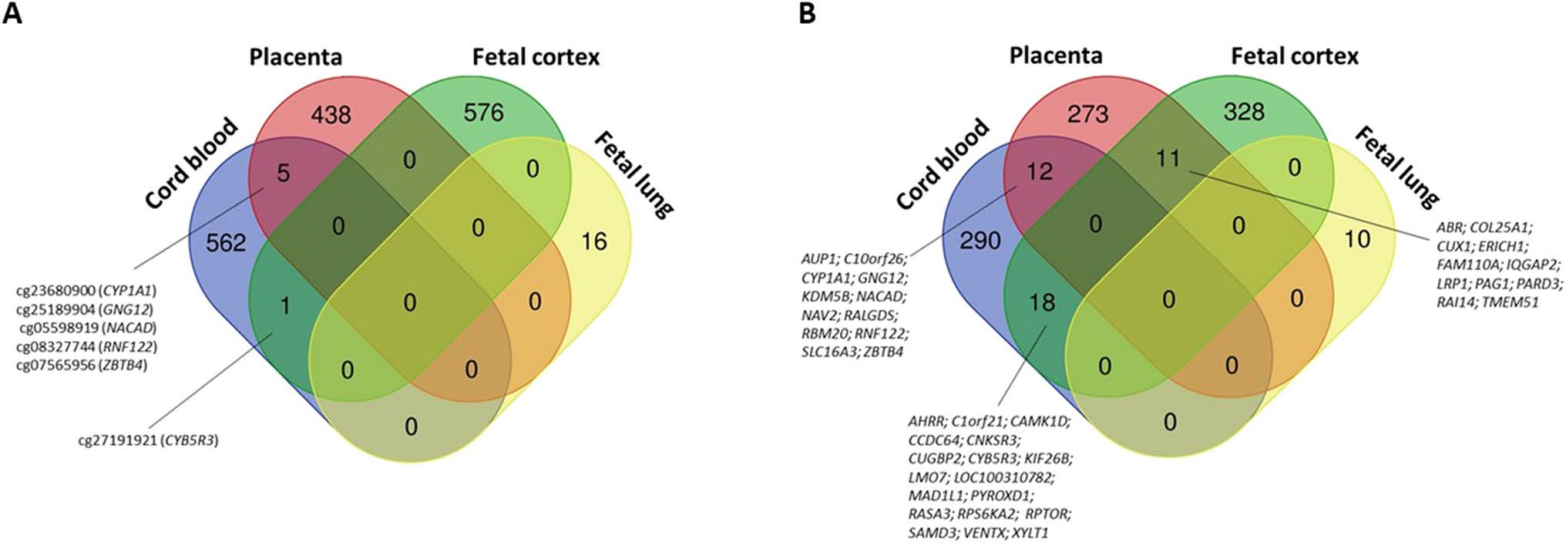
Venn diagram showing the overlap between CpGs (**A**) and genes (**B**) significantly associated with any/sustained maternal smoking during pregnancy in cord blood (Joubert et al. [8••]), placenta (Everson et al. [30••]), fetal cortex (Chatterton et al. [34••]), and fetal lung (Chhabra et al. 35•). The study on buccal epithelium by Breton et al. was not comparable to these studies (the authors assessed repetitive elements and a set of CpGs comprised in the GoldenGate Cancer methylation panel I), and therefore it was not included in the diagram

The overlap of the top CpGs associated with MSDP in the different tissues is shown in Fig. 2A. Five CpGs were common between placenta and cord blood, and one between cord blood and fetal cortex. CpGs in fetal lung did not overlap with CpGs described in any other tissue. Although these observations indicate cell-specific effects of MSDP, we cannot rule out the possibility that different CpGs might be regulating the same genes or pathways, particularly considering that gene expression is often regulated by different CpGs according to the tissue. In fact, when comparing lists of genes mapped to the CpGs significantly associated with MSDP, we found a higher number of overlapping genes: 12 genes overlapped between the placenta and cord blood; 11 genes between fetal cortex and placenta; and 18 genes between cord blood and fetal cortex (Fig. 2B).

### Cell-Type Specificity

Interpreting DNAm changes associated with smoking in the context of tissue cellular heterogeneity is challenging. To answer this, Bauer et al. examined DNAm at 5 smoking-sensitive CpGs in sorted blood cell types [38]. They found that several scenarios were possible: smoking promotes the expansion of a specific cell type with a specific DNAm pattern (i.e., GPR15 + T cells); smoking affects DNAm in specific cells (i.e., hypo-methylation of *GFI1* in granulocytes); and smoking affects DNAm across blood cell types (i.e., hypo-methylation of the cg05575921 CpG in the *AHRR*). In the case of cg19859270, they confirmed that a smoking-induced methylation difference of around 2% was caused by expansion of GPR15 + T cells, involved in inflammation and disease pathology. Thus, even slight DNAm changes in whole blood samples might be of strong biological relevance if attributed to a specific cell type.

However, cell sorting is not always possible in epidemiological settings. To uncover these, there exist reference-based cell deconvolution methods for tissues such as whole blood [39•], cord blood [40•], saliva [41•], or placenta [42•]. Most of the EWAS apply these methods to adjust for cell-type proportions in the statistical model; however, they can also be used to identify cell-type-specific effects. For instance, You et al. found that most highly reproducible smoking-associated hypo-methylation signatures in adults were more prominent in the myeloid lineage [43•].

## Dose and Duration of Maternal Smoking During Pregnancy

The harmful effects of MSDP might be reduced or diminished if the mother quits smoking in the first trimester of pregnancy or if she decreases cigarette consumption. Thus, several studies have explored the effect of these temporal and dose parameters on offspring’s DNAm. Regarding the duration of the exposure, the PACE meta-analyses found that the effects on DNAm were stronger among sustained smoker mothers compared to the group of any smoker mothers, both in cord blood [8••] and placenta [30••]. Consistent findings were found in other studies [13••, 14, 18, 24••].

**Fig. 3:**
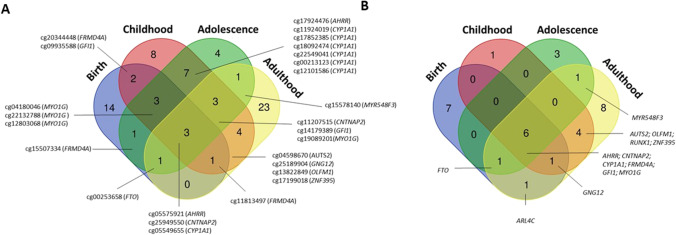
Venn diagram showing the overlap between CpGs (**A**) and genes (**B**) associated with any/sustained maternal smoking during pregnancy showing a dose–response pattern. Studies have been grouped by periods (including from each period the top CpGs/genes found in each of the studies): at birth (Markunas et al. [12]; Richmond et al. [13••]; Monasso et al. [44]), childhood (Vives-Usano et al. [24••]), adolescence (Rauschert et al. [20]), and adulthood (Wiklund et al. [26]). Adulthood CpGs in Wiklund et al. also report dose–response patterns at 16 years

The number of cigarettes smoked per day is also important. Some of the MSDP-sensitive CpGs in cord blood show evidence of a dose-dependent association, with stronger effects with a higher number of cigarettes smoked per day [12, 13••, 44]. These duration and dose-dependent associations of MSDP seem to be maintained in children [24••], adolescents, and adults [20, 26]. The list of CpGs and genes with dose–response across age ranges is shown in Fig. 3.

Studies examining the effect of exposure to second-hand smoke (SHS), which involves lower doses and different exposure routes compared to the exposure through maternal smoking in pregnancy, are inconclusive. Recently, a study reported that exposure to SHS among non-smoking pregnant women was associated with cord blood DNAm changes in several CpGs, some of them overlapping MSDP-sensitive CpGs [45•]. In contrast, other studies did not find any association in child [24••] or adult blood [46]. Similarly, there is low evidence for an association between paternal smoking during pregnancy (PSDP) and cord blood [13••, 44, 47] or placenta DNAm [31, 33•]. The exception is a recent meta-analysis showing associations between PSDP and differential DNAm in offspring’s blood [48•]. However, the study could not determine whether the observed associations were due to SHS exposure during the post-conception period (pregnancy or postnatally) or altered sperm DNAm patterns acquired preconceptionally and transmitted to the offspring.

Regarding preconception exposure, Rousseaux et al. found that placentas of women who quit smoking at least 3 months prior to their pregnancy presented altered DNAm patterns in particular regions of the genome, despite an absence of direct exposure of placentas to tobacco smoke [33•]. If misclassification of the exposure can be ruled out, this suggests the possibility of an acquired epigenetic predisposition, previously described in adipose tissue of former smokers (Tsai et al. 49). In contrast, another study did not find any association of maternal and paternal smoking before pregnancy (MSBP and PSBP) or of grandmother’s smoking during pregnancy (GMSDP) with cord blood DNAm [47].

## Maternal Smoking During Pregnancy and Multi-omics

Biological interpretation of DNAm changes requires annotation of CpGs to genes. Usually, this is done by linking the CpG to the closest gene, but this does not consider long-range chromatin interactions. To address this, some studies base their annotation on *cis* expression quantitative trait methylation (*cis* eQTMs), defined as correlations between DNAm and expression levels of nearby genes. For instance, Everson et al. found that the DNAm at 61.3% of the MSDP-sensitive CpGs were associated with the expression of nearby genes [30••]. The majority (65%) of the eQTMs showed inverse associations (i.e., higher DNAm – lower gene expression). Pathways identified using the eQTM genes differed slightly from those identified using the closest gene annotation. Others have applied similar strategies to identify MSDP responsive genes and pathways in blood [10••]. However, these studies are limited by the small number of individuals in the eQTM analyses, thus having a reduced statistical power. To overcome this, Ruiz-Arenas et al. created a catalog of blood *cis* eQTMs in children, defined as CpG-gene pairs within a 1 Mb window centered at the transcription start site (TSS) (accepted in eLife; https://elifesciences.org/articles/65310). Among the 13 M CpG-gene tests, 39,749 statistically significant eQTMs, representing 21,966 unique CpGs and 8886 unique genes, were found after multiple-testing correction. Most of the associations took place in a distance between the CpG and gene < 250 kb, and 58% showed inverse relationships. Notably, only around half of the eQTM genes could be captured by annotating the CpG to the closest gene. The study also describes low overlap between eQTMs identified in children and adults. However, the authors could not rule out whether this resulted from real biological differences or differences in the study designs. The whole catalog including the 13 M CpG-gene pairs is publicly available at https://helixomics.isglobal.org/.

Multi-omics studies of MSDP are scarce. An exception are the studies by Bauer et al. [22••] and by Vives-Usano et al. 2020 [24••] (Table S1). Vives-Usano et al. analyzed the association of MSDP with multi-omics biomarkers, including blood DNAm, blood gene and miRNA expression, plasma proteins, and serum and urinary metabolites, assessed in children of the Human Early Life Exposome (HELIX) study. MSDP was related to DNAm changes at 18 *loci*, five of which showed an association with the expression of nearby genes. However, no evidence of association was found between MSDP and child blood gene expression, suggesting that the effect of MSDP was more persistent and stronger on DNAm than on gene expression. In line with this, in adults, it has been observed that gene expression levels are recovered to normal levels after smoking cessation faster than DNAm levels are [49]. Regarding other omics, only two child urinary metabolites (alanine and lactate) were associated with MSDP, with low biological plausibility. Metabolites, miRNAs, and transcripts that were previously found altered in current smokers were not among the top statistically significant markers in HELIX [50–53]. The authors also investigated the association of childhood SHS with child molecular signatures. In contrast to what was observed for MSDP, childhood SHS was related to reduced levels of several metabolites (phosphatidylcholines and sphingomyelins) and to increased plasma PAI1 (a protein that inhibits fibrinolysis), both previously described to be altered in current smokers.

Bauer et al. examined the association between DNAm in maternal and offspring’s blood samples through whole‐genome bisulfite sequencing (WGBS), which covers a greater fraction of the genome than array-based methods [22••]. The authors discovered a set of 8409 DMRs associated with MSDP in children, 1404 of which were independent of underlying genetic variants. Child and maternal DMRs were quite distinctive. By analyzing additional data on chromatin histone marks and RNAseq, the authors identified DNAm patterns at enhancers and repressive elements that correlated with transcriptional changes, showing stronger effects later in life than at birth. Two DMRs were validated in a larger sample: a DMR in the *TMEM241* gene and a DMR in a *JNK2* enhancer (in the *GFPT2 gene*). Interestingly, DNAm levels at this enhancer were determined by the combined effect of MSDP and a *cis* mQTL. Finally, loss of DNAm at the *JNK2* enhancer was associated with an increased risk for wheezing, and this was confirmed in a *JNK2* knock-out mouse that had reduced airway inflammation and airway hyperreactivity.

## From Smoking to Health Outcomes: the Role of DNA Methylation

**Fig. 4:**
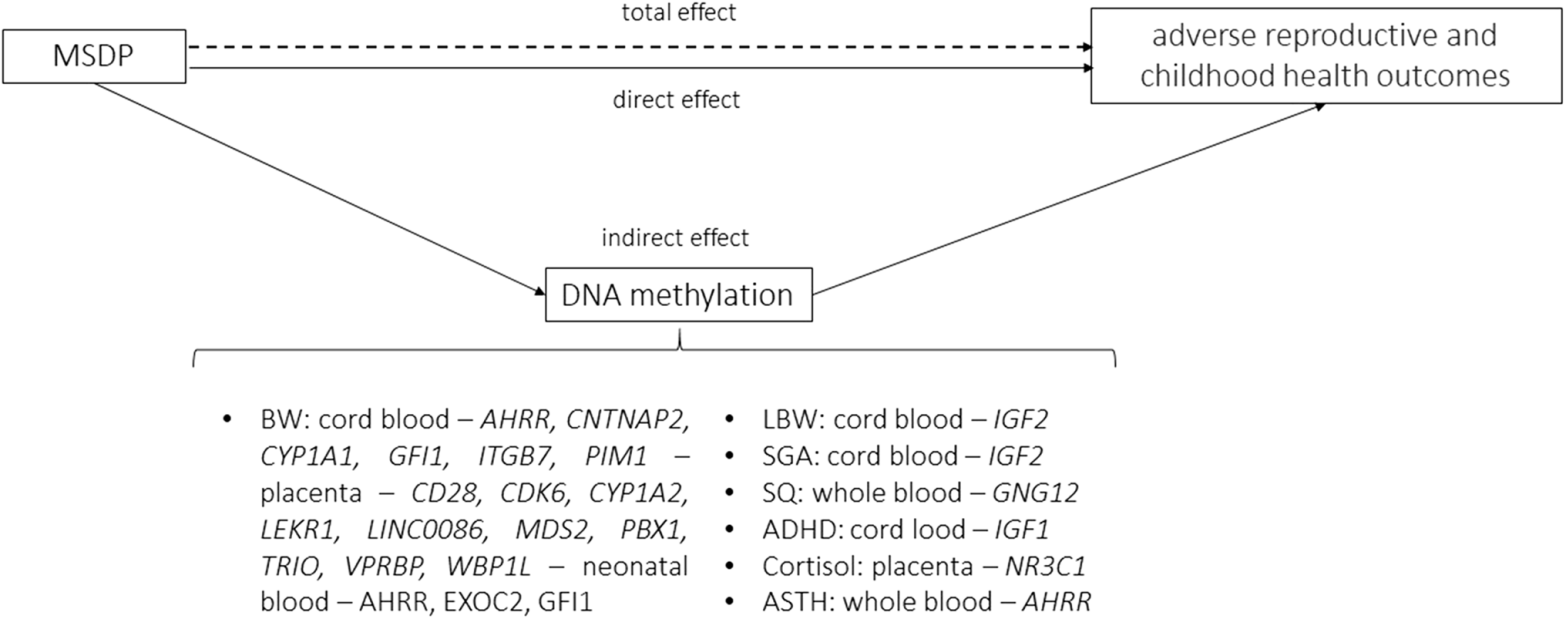
Mediation analysis: the total effect of any/sustained maternal during pregnancy on adverse reproductive and childhood health outcomes is divided in a direct effect and indirect effect through DNA methylation changes. The CpGs/genes for which a mediation effect has been found for different health outcomes and tissues are listed on the right (Murphy et al. [17]; Stroud et al. [61]; Bouwland-Both et al. [19]; Küpers et al. [55]; Morales et al. 31; Valeri et al. [56•]; Witt et al. [57]; Cardenas et al.[32]; Hannon et al. [59]; Neophytou et al. [62]; Wiklund et al. [26]; Miyake et al. [60]; Xu et al. [58]). BW, birth weight; LBW, low birth weight, SGA, small for gestational age; SQ, squizofrenia; ADHD, attention deficit and hyperactivity disorder; ASTH, asthma

It has been suggested that DNAm can mediate the effect of environmental exposures on health outcomes [7]. To explore this, several studies have conducted mediation analyses, which consist of calculating the percentage of the total effect of the exposure that acts through a given mediator factor (indirect effect), and the percentage of the total effect of the exposure unexplained by this same mediator (direct effect) (Fig. 4) [54•].

### Maternal Smoking During Pregnancy, DNA Methylation, and Reproductive Outcomes

Several mediation analyses have been conducted to determine whether the effect of MSDP on birth weight is mediated through DNAm in cord blood [17, 19, 55, 56•, 57, 58], neonate blood [59], or placenta [31, 32] (Table S2).

In cord blood, DNAm at several CpGs of the *GFI1* gene, which is involved in hematopoiesis control, were found to mediate around 18% of the effect of MSDP on birth weight [55]. These results were replicated in cord blood [56•] and neonate blood [59]. Another study described that DNAm at 8 CpGs (close to *AHRR*, *CYP1A1*, and *GFI1* genes) mediated up to 67.8% of the effect of MSDP on birth weight [58]. Lastly, two other studies suggested that the effect of MSDP on the risk of being born with low birth weight or being small for the gestational age could be mediated by DNAm at *IGF2*, an essential gene for fetal growth [17, 19].

Even though blood tissue is easily accessible, there is a concern that it might not be the key tissue for mediating the effect between MSDP and birth weight [55]. In contrast, the placenta is a more plausible tissue in terms of its biology for mediating the effects of MSDP on reproductive outcomes [29]. Indeed, the top CpG in the placenta, cg27402634 (*LEKR1-LINC00086*), was found to explain up to 36% of the effect of MSDP on birth weight, and the CpG cg25585967 (*TRIO*) up to 5% [31]. Another study found seven CpGs likely mediating the effect of MSDP on birth weight, five of which presented an interaction effect between MSDP and DNAm [32].

### Maternal Smoking During Pregnancy, DNA Methylation, and Other Outcomes

Regarding neuropsychological traits, the proportion of the effect of MSDP mediated through blood DNAm is substantial: 48.4% for cord blood DNAm at *GFI1* gene on attention deficit and hyperactivity disorder symptoms [60], and around 30% for adult blood DNAm at the *GNG12* gene on schizophrenia-related outcomes [26]. Another study described that placental DNAm at the *NR3C1* gene could mediate 25% of the effects of MSDP on basal cortisol levels of newborns [61]. Finally, another study found that the odds ratio (OR) for the indirect effect of MSDP on asthma mediated through blood DNAm at the cg05575921 CpG (*AHRR*) was 1.18, being the OR of the total effect 1.48 [62].

### Limitations of Mediation Analyses and Causal Inference

Findings from a mediation analysis must be interpreted with caution because they have several limitations. First, they give biased results when the mediator (i.e., DNAm) captures the exposure (i.e., MSDP) with less error than the method used to assess the exposure itself [56•], and when there is mediator-outcome confounding, exposure-mediator interaction or poorly specified models [32, 54•]. Second, they only prove a statistical relationship between the factors (exposure-mediator-outcome), but not a causal link. For causal inference, triangulation, which consists in obtaining more reliable answers to research questions by integrating results from different study designs, is essential [63]. Thus, EWAS findings from observational studies should be complemented with Mendelian randomization (MR) analyses, clinical trials, and validation in animal models, when possible.

Mendelian randomization is a statistical method that uses genetic variants that influence DNA methylation (mQTLs) as instrumental variables to evaluate the causal link between an exposure (i.e., DNAm) and an outcome [64•]. For instance, Morales et al. found suggestive evidence that decreased placental DNAm levels at the CpG cg27402634 (*LEKR1-LINC00086*) lead to reduced birth weight [31]. Similarly, Wiklund et al. suggested that MSDP is associated with an increased risk of schizophrenia by a decrease of placental DNAm levels at the CpG cg25189904 (*GNG12*) [26]. MR analyses of DNAm or other molecular traits require public databases of molecular quantitative trait *loci* (QTLs) to identify the instrumental variables. While databases of expression QTL are publicly available for diverse tissues at websites such as the one by the Genotype-Tissue Expression (Gtex) project (https://gtexportal.org/home/), there are fewer, ancestry dependent, and generally smaller, public databases for mQTLs [65•, 66, 67].

## Epigenetic Scores to Predict Past Exposure to Tobacco Smoke During Pregnancy

DNAm patterns can predict past exposures to MSDP, which can overcome missing, incomplete, or inaccurate data on MSDP. The first epigenetic score of MDSP for cord blood, which included 28 CpGs, was developed by Reese et al. using an iterative logistic lasso cross-validation procedure [68]. The area under the curve (AUC) value, which is calculated according to the specificity and sensitivity of the score, was 0.90 for the testing cohort. A subsequent score for adult blood derived using the coefficients of 19 CpGs associated with MSPD in child blood from the PACE meta-analysis [8••], had moderate accuracy (AUC = 0.72) [69]. Recently, Rauschert et al. developed an epigenetic score for adolescent and adult blood containing 204 CpGs which were selected using the elastic net regression method [70••]. The score had AUC values > 0.80 for the testing cohorts and in these cohorts it outperformed the previous ones.

Finally, Blostein et al. developed an epigenetic smoking score for children and adolescents based on saliva DNAm (https://www.medrxiv.org/content/10.1101/2021.11.30.21267020v1.full). Even using weights of 6,074 CpGs from cord blood [8••], the AUCs were 0.78 at the age of 9 and 0.77 at the age of 15. Moreover, the authors found that the score was quite portable across ancestry groups.

## Conclusions

The existing literature supports a significant and consistent impact of MSDP on the offspring’s epigenome at biologically relevant genes across important tissues such as cord blood and placenta. This information has been successfully used to predict past exposure to MSDP through epigenetic scores. Despite this evidence, there are still some gaps to be addressed, some of which have already been highlighted by a previous review [71]. First, while it is clear that the duration of MSDP affects DNAm levels, the evidence for maternal or paternal smoking during the preconception period is less consistent. Moreover, although MSPD dose–response patterns have been described in several CpGs, the effect of low doses such as for SHS is difficult to address. Similarly, more studies are needed to confirm the implications of the observed persistent effects in the epigenome, addressing the existence of cell memory mechanisms and the potential association with increased vulnerability to similar exposures later in life or future generations. Second, there is a need to investigate whether the effects of MSDP are consistent between sexes and ancestry groups, and the effect modification of genetic variants or environmental factors should be considered more systematically. Third, translation of DNAm changes in certain CpGs into biological pathways requires additional deeper multi-omics data, especially transcriptomic data. Biological interpretation is, in addition, complicated due to limitations in tissue accessibility and cell-type specificity. Studies from abortions on fetal organs might offer more direct knowledge on otherwise inaccessible target organs. In turn, cell-type specificity can be addressed by conducting cell sorting, single-cell DNAm studies, or otherwise, by applying cell deconvolution methods. Finally, proving causal links between DNAm and health outcomes requires triangulation of findings from different study designs: observational, MR, and animal models. Overall, despite most of the findings reviewed here are robust and consistent, further investigations are guaranteed to provide a more comprehensive understanding of MSDP impact on the offspring epigenome by combining data obtained using newer technical approaches and multiple study designs.

## Supplementary Information

Below is the link to the electronic supplementary material.

Supplementary file1 (XLSX 88 KB)

## Abbreviations

ALSPAC: Avon Longitudinal Study of Parents and Children
AUC: Area under the curve
CpG: Cytosine nucleotide followed by a guanine nucleotide
DMR: Differentially methylated region
DNAm: DNA methylation
EPIC: Infinium HumanMethylationEPIC BeadChip—Illumina
eQTL: Expression quantitative trait locus
EWAS: Epigenome-wide association study
FDR: False discovery rate
GMSDP: Grandmother smoking during pregnancy
HELIX: Human Early Life Exposome
mQTL: Methylation quantitative trait locus
MR: Mendelian randomization
MSBF: Maternal smoking before pregnancy
MSDP: Maternal smoking during pregnancy
PACE: Pregnancy And Childhood Epigenetics
PBMCs: Peripheral blood mononuclear cells
PMD: Partially methylated domains
PSBF: Paternal smoking before pregnancy
PSDP: Paternal smoking during pregnancy
QTL: Quantitative trait locus
SHS: Second-hand smoke
TF: Transcription factors
TSS: Transcription start site
WGBS: Whole-genome bisulfite sequencing
27 K: Infinium HumanMethylation27 BeadChip – Illumina
450 K: Infinium HumanMethylation450 BeadChip—Illumina
